# ADDRESSING THE NURSING WORKFORCE GAP IN SKILLED NURSING FACILITIES WITH AN AGE-FRIENDLY CURRICULUM

**DOI:** 10.1093/geroni/igae098.3560

**Published:** 2024-12-31

**Authors:** Diane Brown, Sue Fosnight, Jennifer Drost, Mary Gergis, Cynthia Hovland, Margaret Sanders, Denise Kropp, Michele Gareri

**Affiliations:** University of Akron, Akron, Ohio, United States; Summa Health, Akron, Ohio, United States; Summa Health, Akron, Ohio, United States; Cleveland State University, Cleveland, Ohio, United States; Cleveland State University, Cleveland, Ohio, United States; Northeast Ohio College of Medicine, Rootstown, Ohio, United States; Northeast Ohio College of Medicine, Rootstown, Ohio, United States; Summa Health, Akron, Ohio, United States

## Abstract

Skilled nursing home quality and staffing are positively correlated. Staffing shortages have been a long-standing problem however, COVID-19 exacerbated the issue. Currently, more than one in four US nursing homes report staff shortages, especially those in direct care to residents, nurse aides and nurses. To encourage workforce growth, a 12-module virtual curriculum was developed including Age-Friendly Health Systems care, interprofessional teamwork, and other relevant topics. Each module included a recorded didactic, pre-post module quizzes, discussion boards, and supplemental videos and articles. Trainees also spent 10 hours shadowing the interprofessional team in a skilled nursing facility. Two cohorts of undergraduate senior nursing students (n=18, and n=35), nurse assistants in training (n=9, cohort 1), and social work students (n=9, cohort 2) have been trained. Outcome measure were pre and post-education using surveys: Nursing Home Attitude, PES-NWI work environment, and ISVS-9 for teamwork attitude. Results of the first nursing cohort rated the highest nursing home attitude items: ability to provide comfort, flexible scheduling and spending time with clients with the lowest rating for working with persons with dementia. The PES-NWI survey showed highest concern for teamwork, adequate staffing and supportive administration with the lowest concern related to use of nursing diagnoses. The ISVS showed similarities pre and post with highest scores for client involvement in care and importance to work as a team, and lowest for comfort leading a team. Administrators of skilled nursing and educators can use this information to close the gap on improving interest in this setting for new graduates.

